# Inoculation frequency and maize genotype influence plant growth-promoting effects of soil bacteria under low nitrogen conditions

**DOI:** 10.3389/fpls.2025.1637156

**Published:** 2025-09-12

**Authors:** Lexie R. Foster, Jinliang Yang, Jean-Jack M. Riethoven, Hussnain Mukhtar, Daniel P. Schachtman

**Affiliations:** ^1^ Nebraska Center for Biotechnology, University of Nebraska-Lincoln, Lincoln, NE, United States; ^2^ School of Biological Sciences, University of Nebraska-Lincoln, Lincoln, NE, United States; ^3^ Department of Agronomy and Horticulture, University of Nebraska-Lincoln, Lincoln, NE, United States; ^4^ Center for Plant Science Innovation, University of Nebraska-Lincoln, Lincoln, NE, United States

**Keywords:** nitrogen-use efficiency, plant-growth-promoting bacteria, rhizosphere, maize genotypes, bacterial inoculation, biostimulants

## Abstract

Global agriculture relies heavily on the use of synthetic nitrogen fertilizer to meet the current global food demand. Unfortunately, the average nitrogen-use efficiency (NUE) of maize (*Zea mays ssp. mays*) is as low as 50%. Improving the NUE of maize is essential for feeding the ever-increasing world population while also decreasing the negative environmental impacts of nitrogen fertilizer due to runoff and volatilization. Harnessing the symbiotic relationship between plants and soil microorganisms may be one method for increasing the NUE in crops such as maize. In the present study, a set of potentially beneficial bacterial species chosen based on genetic information from the host was investigated for their ability to improve NUE-related traits in maize grown under nitrogen-deficient conditions. This was carried out through non-repeated and repeated bacterial inoculations using different maize genotypes. We identified several growth-promoting bacterial isolates and observed a significant interaction between the bacterial isolates and the maize genotype, suggesting a strong interaction between the host genetics and the effects of bacterial isolates. In addition, our results showed a significant growth response to repeated inoculations with a beneficial bacterial isolate. In summary, when evaluating the plant-growth-promoting effects of a bacterial species, it is essential to consider the interaction between host plant genotype and bacterial isolate. In addition, when inoculating with bacterial isolates, multiple inoculations appear to be more effective than a single inoculation after bacterial seed priming.

## 1 Introduction

To meet the growing global food demand, there has been a significant increase in nitrogen (N) fertilizer use over the past century, which has directly influenced the rise in yields over the same time period (Cao et al., 2018). U.S. maize yields increased from 1930 to 1960 with an average gain of 63 kg^-1^ ha^-1^ and an average gain of 110 kg^-1^ ha^-1^ from 1960 to 2000 (Woli et al., 2018). From 1940 to 2015, N fertilizer usage also increased from 0.28 g N m^-2^ y^-1^ to 9.54 g N m^-2^ y^-1^ (Cao et al., 2018), which was a major factor responsible for increased maize yields. The increase in N fertilizer application and the genetic improvement of maize (Assefa et al., 2012) have been the key factors driving the upward trajectory of yield in maize. While N is essential for high yields, fertilization of agricultural land contributes to approximately 53 percent of the global anthropogenic emission of nitrous oxide (N_2_O) (Denman et al., 2007). The N_2_O released from N fertilization is particularly a concern for global climate change as it has a warming potential 298 times greater than CO_2_ (Lan et al., 2022). With the increasing application of N fertilizer, maize (*Zea mays*) crops only use between 25–50 percent of the applied N (Javed et al., 2022). An increase in the nitrogen-use efficiency (NUE) of global crops, such as maize, is important not only for decreasing the negative environmental and human health impacts of N fertilization but also for maintaining high crop yields.

Plants utilize N as either NH_4_^+^ or, more commonly, NO_3_^-^ (Novoa and Loomis, 1981). Soil NO_3_^-^ can be sensed by plant roots, allowing for the activation of NO_3_^-^ transporters for uptake of NO_3_^-^ (Aluko et al., 2023). Plants are equipped with various mechanisms for adapting to growth under N-deficient conditions. For example, the mechanism called stress-initiated nitrate allocation to roots (SINAR) is coordinated in part by the nitrate transporters *NRT1.5* and *NRT1.8* (Zhang et al., 2014). Under nitrate sufficient conditions *NRT1.8* unloads nitrate into leaves and NRT1.5 loads nitrate into the xylem for long distance transport to leaves. However, under nitrate-deficient conditions, NRT1.5 gene expression is down-regulated, and NRT1.8 expression is increased in roots, leading to the unloading of nitrate from the xylem into the roots (Li et al., 2010) which increases root elongation to maximize N acquisition from the soil (Chun et al., 2005). In addition, the inducible high-affinity transport systems (IHATS) transporters are activated when NO_3_^-^ concentrations are low in the soil (Crawford and Glass, 1998) to enable uptake at very low concentrations of available nitrate. Another strategy plants use to adapt to N-deficient conditions is to increase their production of indole-3-acetic acid (IAA) (Lv et al., 2021; Zhang et al., 2023), as IAA has been shown to increase root elongation, which would aid in N uptake (Zhang et al., 2023). These strategies for adaptation to N-deficient conditions and for increasing nutrient acquisition are important for maintaining yields (Abdel-Lattif et al., 2019; Raza and Farmaha, 2022).

Microorganisms in soils may also play important roles in supplying plants with N and in regulating the forms of N that are available to plants. Soils harbor a diverse variety of bacteria, archaea, fungi, and viruses, collectively making up the soil microbiome (Bardgett and van der Putten, 2014). The rhizosphere microbial communities are in direct proximity to plant roots, and are influenced by both plant uptake of nutrients and the exudation of compounds from roots, leading to an intimate host plant and microbe interaction (Trivedi et al., 2020; Chai and Schachtman, 2022b; Lopes and Schachtman, 2023b). These chemical compounds, referred to as root exudates, can influence the diversity and composition of rhizosphere microbial communities (Bertin et al., 2003). Therefore, when a plant encounters a stressful environment due to a nutrient deficiency, such as low N, it may alter its exudate composition and thereby attract a community of microbes that will aid in root growth, thereby increasing nutrient acquisition (Coskun et al., 2017).

Certain microbial species play major roles in the nitrogen cycle, such as nitrification which may benefit crop growth (Franche et al., 2009) and also lead to losses of N from soils. Nitrifying bacteria in the soil convert NH_3_ into NO_3_^-^, which is a form of N that plants readily utilize (Werner and Newton, 2005). Key members in the nitrification process include ammonia-oxidizing bacteria (AOB), such as *Nitrosospira* and *Nitrosomonas*, as well as nitrite-oxidizing bacteria (NOB), including the phyla Proteobacteria, Chloroflexi, Nitrospina, and Nitrospira (Clark et al., 2021). As part of the soil nitrogen cycle, bacteria also carry out denitrification, a process in which NO_3_^-^ is reduced to N_2_O or N gas (Martienssen and Schöps, 1999) and subsequently lost to the environment. Another way in which some plant species can acquire N is from a symbiotic relationship with a bacteria that forms nodules on roots and that fix N from atmospheric N_2_ for use by plants (Soumare et al., 2020). There are also free-living bacterial species in the soil that fix N which may be taken up by plants and some of these include *Azospirillum spp., Acetobacter diazotrophicus, Herbaspirillum seropedicae, Azoarcus* spp., and *Aztobacter* (Steenhoudt and Vanderleyden, 2000). Because these free-living N fixing bacteria occur naturally within the soil, they can be further harnessed to provide plants with N through the design, formulation, and application of microbial inoculants.

Countries including Brazil and Argentina have adopted the use of microbial inoculants such as *Azospirillum* strains in their agricultural practices. *Azospirillum* species, such as *A. brasilense* and *A. lipoferum*, have been identified as plant-growth promoting bacteria (PGPB) in maize and wheat plants (Hungria et al., 2010). Other studies have confirmed the use of *Azospirillum* spp. as PGPB in maize (Galindo et al., 2019, Galindo et al., 2022; Hungria et al., 2022). In 1996 in Argentina, the first registered inoculant was developed using *A. brasilense* Az39, followed by the first registered inoculant in Brazil in 2010, developed with the *A. brasilense* Ab-V5 and Ab-V6 strains (Cassan et al., 2020). In addition to N fixation (Han and New, 1998) there have been other reports of plant-growth-promoting characteristics of *Azospirillum* spp. including the production of phytohormones, such as IAA (Tien et al., 1979) and gibberellins (Bottini et al., 1989). While identifying a growth-promoting function of a microbe is critical when developing a microbial inoculant, it may also be necessary to account for the interaction between the microbe and the plant genotype being inoculated (Malacrino et al., 2022). Only a few studies have demonstrated the interaction between microbial inoculants and plant genotypes (Andreote et al., 2010; French et al., 2020; Chai et al., 2021).

Past crop breeding efforts have neglected the role of the plant microbiome in crop improvement. To better integrate the microbiome with plant genetics, a previous study (Meier et al., 2022) identified a group of amplicon sequence variants (ASVs) in the rhizosphere microbiome of inbred maize plants grown under both optimal N and N-deficient conditions. These bacterial ASVs were correlated with plant traits, such as canopy coverage and agronomic traits, as well as associated with the maize genetic loci, suggesting a strong correlation between maize genetics, bacterial species in the rhizosphere, and plant performance. Based on these previous results (Meier et al., 2022) that identified ASVs associated with maize loci, we hypothesized that these bacterial ASVs may enhance maize growth in N-deficient conditions. To test this hypothesis, we used these ASVs to identify and then test 63 bacterial isolates identified from an existing culture collection. These isolates were then tested in a series of controlled environment chamber experiments to determine how their introduction assisted in the growth of maize under low N conditions. The results of this study provide insight into the potential of using culture-independent microbiome data, coupled with plant genetic information, for selecting growth-promoting isolates from a culture collection. Additionally, our results shed light on the impact of using a repeated inoculation method for applying PGPB and highlight the importance of considering the interaction between a PGPB and the plant genotype being inoculated.

## 2 Materials and methods

### 2.1 Identification of potentially beneficial isolates

#### 2.1.1 Field-based ASV identification

Amplicon sequence variants (ASVs) of soil microbes were identified from 3,313 rhizosphere samples based on their associations with maize genes (Meier et al., 2022). ASVs that were observed across two years of sampling were identified from paired-end 16S sequencing reads collected from the samples. That analysis showed the maize genome likely underwent negative or positive selection to favor specific microbial taxa (referred to as rhizobiome traits) by removing deleterious alleles or duplicating desirable alleles. We hypothesized that plant-associated ASVs possibly contributed to increasing plant fitness because of their association with maize genes.

#### 2.1.2 Culture collection description

The ASVs from that study were then screened against the 16S sequences from a culture collection that was developed over several years and contains plant and soil derived bacterial isolates from multiple regions of crop and grassland soils. The collection of approximately 4500 isolates was cultured from soil, rhizosphere and roots. Approximately 2700 of the isolates have had all or part of their 16S gene sequenced. That sequence was used to search databases for taxonomic assignments. In addition, 472 isolates have genome sequences that were generated by the Joint Genome Institute (Berkeley, CA) using Illumina sequencing. Those sequences and the annotations are contained in IMG/M database (https://img.jgi.doe.gov/). The agricultural fields were located near the following Nebraska cities: North Platte, Brule, Mead, North Bend, Valley. Samples from grasslands were collected in the Sandhills and that sampling was previously described (Lopes et al., 2021). Root and rhizosphere samples were collected from a range of different plant species including corn, sorghum, soybean and the grasses as described (Lopes et al., 2021).

#### 2.1.3 Sequence alignment and matching process

To match the 724 ASVs identified from the field-based study (Meier et al., 2022) with the 16S data from the culture collection, four BLAST (Camacho et al., 2009) databases were created from the culture collection data that contained the merged, 27F, 515F, and 515R sequences. The field-based ASVs were aligned via ungapped BLAST against each database with default parameters. A custom Perl script was then used to scan all BLAST output files and collate information by ASV ID for the best alignment for individual subject IDs (i.e., microbial database sequence ID), with a requirement that the difference between the alignment length and the query (ASV) length cannot be larger than two nucleotides. Alignments to the merged sequences have preference over 27F, and 27F alignments have preference over 515F, and so forth. Of the field-identified ASVs, 629 (87%) aligned to the in-house culture collection data. The matching ASVs corresponded to 63 bacterial isolates (**Supplementary Table 2**) from the culture collection described above. The bacterial isolate sequences were between 100% identity to 95.205%, similar to the field-identified ASVs (**Supplementary Table 1**). Of these 63 isolates, 35 have draft genome sequences that may be found in the JGI IMG/M database (**Supplementary Table 2**). The isolates from the culture collection were then revived on nutrient-containing plates from glycerol stocks stored at -80°C and used in the experiments described below.

### 2.2 Maize seedling bacterial inoculation

Mo17 inbred maize was selected for the initial screening of the 63 bacterial isolates, the 14-day validation experiments, and as one of the three maize genotypes in the non-repeated and repeated inoculation experiments. The two other maize genotypes selected for the non-repeated inoculation experiment were NSL 30867 and PI 606768 and for the repeated inoculation experiment NSL 30867 and Ames 27065 were used. These maize genotypes were selected based on their root exudation of sugars (Lopes et al., 2022). The maize genotypes PI 606768 and Ames 27065 exuded relatively high concentrations of total sugars, while the maize genotype NSL 30867 exuded low concentrations of total sugars.

For all experiments, maize seeds were surface sterilized for 48 hours using chlorine gas, produced by mixing 4 mL concentrated hydrochloric acid (HCl) with 100 mL bleach in a desiccator. The chlorine gas was replaced after 24-hours. PI 606768 was sterilized for only 24 hours utilizing the same protocol, as a 48-hour sterilization decreased the germination rate of this maize genotype. Following the surface-sterilization, the seed was placed in aerated, autoclaved (20 minutes sterilization, at 121°C, 10 minutes drying) Milli-Q water to imbibe for 12–24 hours at room temperature (24°C). After imbibing, the seeds were placed in petri dishes lined with sterilized moist filter paper. The seeds were then sprayed with Captan (0.2%) to control fungal growth. The petri dishes were sealed with micropore tape and placed at 30°C in the dark for 24–48 hours until the seeds germinated.

Based on the information in the culture collection database the 63 bacterial isolates were inoculated and grown in one of the following media types depending on how they were originally isolated: yeast extract-peptone-dextrose (YPD), yeast mannitol agar (YMA), trypticase soy agar (TSA), reasoner’s 2A agar (R2A), or Ashby’s N-free medium (Stella and Suhaimi, 2010). Bacterial isolates were plated from glycerol stocks that were stored in a -80°C freezer. Germinated maize seeds were placed in a sterile petri dish when the root was about 1 cm long and approximately 8 mL of the liquid bacterial culture (OD_600_ of 1) was added. The petri dish was sealed with micropore tape and placed on the rotary shaker at 80 rpm for 12 hours at room temperature (24°C). This inoculation method is referred to as a seedling priming (Chai et al., 2022a). Mock-inoculated germinated seeds were treated similarly by placing them in a sterile R2A medium without any added bacteria for 12 hours and were used for the uninoculated controls grown in low and high N.

For all experiments, germinated maize seeds were inoculated using a seedling priming method with only one of the 63 bacterial isolates for each treatment. The sterility of the system ensured that only one bacterial isolate was tested in each trial for growth promoting effects on maize grown under low N conditions.

### 2.3 Screening of the 63 bacterial isolates and 14-day validation experiment

The screening of the 63 bacterial isolates for their plant-growth-promoting activity was carried out in eight rounds because of space limitations in the growth chamber. Initially the screening was done with n = 3 replicate Mo17 plants and then increased to n = 8 replicates per isolate tested. In each 14-day round, an uninoculated replicated set of maize plants was grown in N-deficient conditions (low N control) and N-sufficient conditions (high N control) in sterile growth bags. The germinated maize seed was inoculated with each of the 63 isolates and grown in pots (11 cm in height, 13 cm in diameter) containing 500g of calcined clay for 14 days. To ensure sterility the pots containing the calcined clay were autoclaved 3 times (25 minutes sterilization at 121°C, 10 minutes drying). A pot was then placed into a sterile growth bag (*Nasco-Whirl-PAK*) with an *AeraSeal* film placed over a hole cut in the bags to allow for gas exchange. This allowed for a sterile growth system. A volume of 450 mL of a modified (Schachtman and Munns, 1992) Hoagland’s nutrient solution with either sufficient N (14.5 mM NO_3_, 1 mM NH_4_) or with 10% NO_3_ (1.45 mM) and 50% NH_4_ (0.5 mM) was added to the calcined clay, bringing the system to 90% maximum soil water holding capacity (SWHC). The Hoagland solution was prepared with autoclaved Milli-Q water. The N-sufficient control plants (high N control) were given 450 mL full-strength Hoagland solution (15.50 mM N). Following the Hoagland solution, an inoculated maize seed was sown into the calcined clay at a 1-inch depth and then grown in a growth chamber. The planting was done in a laminar flow hood to ensure the presence of only the desired bacterial isolate. The conditions of the growth chamber were kept at 26°C during the day and 18°C during the night, with a 16-hour light period, throughout the course of all experiments. The average light intensity of the growth chamber was 350 μmol m^-2^ s^-1^. For the screening and 14-day validation experiments, no additional water or nutrients were added throughout the 14-day growth period.

Following the screening of the 63 isolates, none of the isolates imparted a statistically significant growth increase to Mo17 under low N conditions. But to test what appeared to be isolates that increased growth under low N, albeit not significantly, we chose 15 isolates to be tested again in another 14-day experiment. Plants were grown and inoculated as described for the screening experiment. However, the design of this experiment was improved using a randomized complete block design (RCBD). Due to space limitations in the growth chamber, the 15 bacterial isolates were divided into two groups for two 14-day experiments. There were 8 blocks in total, with each bacterial isolate-inoculated plant replicated per block along with two uninoculated control plants at low N and two uninoculated plants at high N. Due to some of the plants not emerging successfully, each bacterial isolate-inoculated plant had 7–8 replicates, with the low N control plants having 16 replicates in both 14-day validation experiments (n = 32 total for the low N control).

### 2.4 Non-repeated inoculation experiment

In the non-repeated inoculation experiment, pots (19 cm in height, 15 cm in diameter) were filled with 2/3 peat and 1/3 fine vermiculite mixture. This soil mixture was almost completely depleted of N. The pots containing the peat-vermiculite mixture were autoclaved three times (20 minutes sterilization, at 121°C, 10 minutes drying), to ensure sterility of the growth medium.

Three bacterial isolates were selected from the 15 tested in the 14-day validation experiment. One of these isolates increased the growth of Mo17 on low N significantly, while two others boosted growth, but not significantly. A 3 x 5 factorial experimental design was implemented. Three different maize genotypes were used as well as five treatments which included three bacterial isolates and the uninoculated maize in low N and in high N as controls. The treatments were arranged into 9 blocks, with each treatment containing one of the three maize genotypes and tested with three bacterial isolates and two controls in one replicate per block (n = 9). Due to poorer emergence rates, the PI 606768 low N control treatment only had 7 replicates (n = 7) and the 4589- inoculated PI 606768 treatment group only had 4 replicates (n = 4). The three maize genotypes used were Mo17, PI 606768, and NSL 30867.

Germinated seeds were treated with bacterial cultures as described for the screening experiment. Two inoculated maize seeds were sown into each pot. On the seventh day cultures for each of the three bacterial isolates was added to each pot. Cultures were resuspended in low N sterile Hoagland’s solution and adjusted to an OD_600_ of 0.002 and ten mls were added to each pot. The pots containing the seeds inoculated with bacterial isolates, as well as the N-deficient uninoculated plants (low N controls) were given a half-strength Hoagland nutrient solution at the time of planting, up to 85% SWHC. The high N control plants were treated with a full-strength Hoagland nutrient solution at the time of planting. Upon planting the pots were covered with plastic saucers and placed in the growth chamber. The planting process took place inside the laminar flow hood. The plants were monitored and watered to approximately 85% SWHC with low N nutrient solution twice per week throughout the 35-day growth period. At the same timepoints, full-strength Hoagland nutrient solution was given to the high N control plants.

To track the growth of the plants at 14, 21, and 28 days after planting, a phenotyping system was utilized to measure the aboveground biomass of the plants using images. The key components of this phenotyping system include a precision rotation platform (Newport Cooperation, Irvine, California), an RGB camera (Logitech BRIO 1080p Webcam) and a computer. A graphic-based LabVIEW program was used to operate the system and automatically capture multiple side views from all angles of the targeted plant within seconds. The phenotyping system captured 8 images at multiple angles over 360°. Using *R* (R Core Team, 2023), the aboveground biomass of each plant was accurately measured through the processing of the eight images. At 14, 21, and 28 days after planting, the plants were photographed to measure their shoot biomass. Upon the completion of the collection, the images were processed in batch from a specified directory using the “EBImage” (Pau et al., 2010), “imager” (Barthelmé and Tschumperlé, 2019), and “raster” (Hijmans, 2025) libraries in R, where each image was manually cropped and converted to a grayscale matrix based on the green channel. A binary raster grid was then generated by thresholding pixel values and applying a distance-based mask to isolate central features, with the processed binary images saved to a new directory. Edge detection was subsequently used to extract morphological traits such as shoot width, pixel-based biomass, and occupancy ratio. From the repeated inoculation experiment, a correlation coefficient between the 27-day photographed biomass measurements and the dry shoot weights of the 28-day old plants was developed in *MatLab* (The MathWorks Inc., 2022).

### 2.5 Repeated inoculation experiment

**Figure 1:**
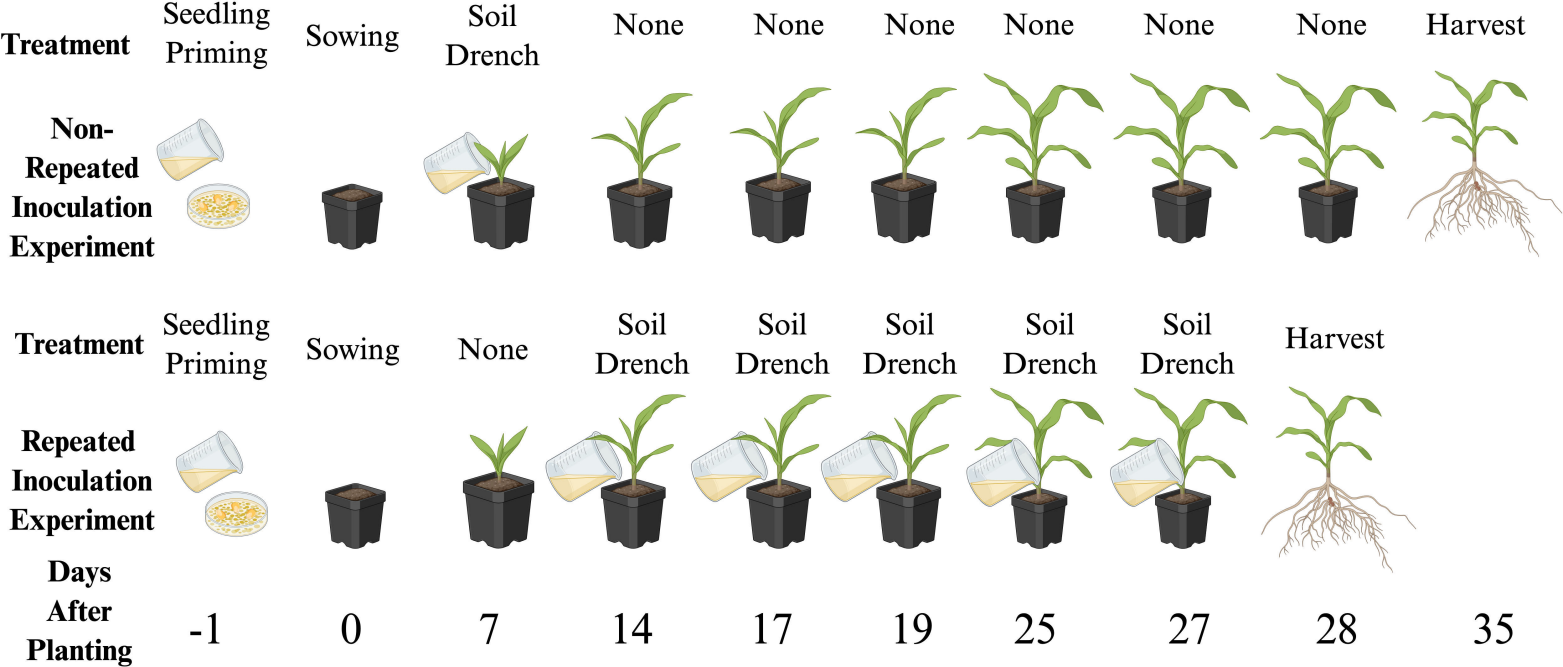
Inoculation schedule for the non-repeated and repeated inoculation experiments. In the non-repeated inoculation experiment, the maize plants were inoculated with the seedling priming technique the day before planting, given a soil drench inoculation 7 days after planting, and harvested 35 days after planting. For the repeated inoculation experiment, the maize plants were inoculated with the seedling priming technique the day before planting and then repeated soil drench inoculations were performed at 14, 17, 19, 25, and 27 days after planting and harvested 28 days after planting. Low N nutrient solution was added to both experiments twice per week. Both experiments were set up as randomized complete blocks (n = 9 blocks) and analyzed using a linear mixed model. Created in BioRender. Foster (2025). https://BioRender.com/s0lhnex.

The repeated inoculation experiment used the same experimental design as the non-repeated experiment, except that the inoculation protocol was altered. The inoculation protocol consisted of seedling priming followed by 5 drenches with bacteria using the same method as described above (**Figure 1**). Soil drenching with a bacterial inoculant was done 14, 17, 19, 25, and 27 days after planting. The same three bacterial isolates, 111 (*Arthrobacter* sp.), 730 (*Pseudomonas kribbensis*), and 4589 (*Sphingomonas* sp.), were used. The three maize genotypes used in the repeated inoculation experiment were Mo17, Ames 27065, and NSL 30867. This repeated inoculation protocol was tested since the growth-promoting properties of bacteria may vary based on the concentration and recurrence of inoculation (Papin et al., 2025). The same randomized complete block design was used as described above. There were 9 blocks in the experimental design, with 1 replicate of each treatment in each block. Due to differences in germination rates, most of the treatment groups had 8 replicates (n = 8), except the NSL 20867 inoculated with isolate 730 (n = 9), NSL 30867 low N control (n = 9), Ames 27065 low N control (n = 7), and Ames 27065 inoculated with isolate 730 (n = 6). The same planting, watering, and growing conditions were used, as described above.

To non-destructively measure the growth of the plants over the course of the 28-days, the same phenotyping system was utilized for phenotyping at the 14-, 21-, and 27-day timepoints. The images were analyzed using the same protocol as the non-repeated inoculation experiment.

### 2.6 Phylogenetic analysis and visualization

63 of the isolates were analyzed. The evolutionary history was inferred using the Neighbor-Joining method (Saitou and Nei, 1987). The bootstrap consensus tree inferred from 1,000 replicates is taken to represent the evolutionary history of the taxa analyzed (Felsenstein, 1985). Branches corresponding to partitions reproduced in less than 50% bootstrap replicates are collapsed. The evolutionary distances were computed using the p-distance method (Nei and Kumar, 2000) and are in the units of the number of base differences per site. The analytical procedure encompassed 63 nucleotide sequences. The complete deletion option was applied to eliminate positions containing gaps and missing data. Evolutionary analyses were conducted in MEGA12 (Stecher et al., 2020; Kumar et al., 2024) utilizing up to 8 parallel computing threads.

### 2.7 Statistical analysis

The statistical analysis of the initial screening was carried out in *R* (R Core Team, 2023). A one-way ANOVA test was performed on the dry shoot weight of the plants in the screening assay. Inoculated plants that appeared to offer a growth effect on the dry shoot weight compared to the uninoculated low N control plants were selected for the 14-day validation experiment.

The statistical analyses of the 14-day, non-repeated and repeated growth experiments were carried out in *R* and *SAS* software (SAS Institute Inc., 2023). SAS software, Version 9.4 M8. Cary, NC: SAS Institute Inc.). A linear mixed model analysis was performed to determine if the dry shoot weight of the maize grown with each bacterial isolate was significantly higher than the dry shoot weight of the maize grown in low N conditions that lacked added bacteria. Within the linear mixed model, LS-means, also known as model-adjusted means were calculated for each treatment group. A Tukey HSD *post-hoc* analysis was performed to determine which specific treatment groups were significantly different from one another.

**Figure 2:**
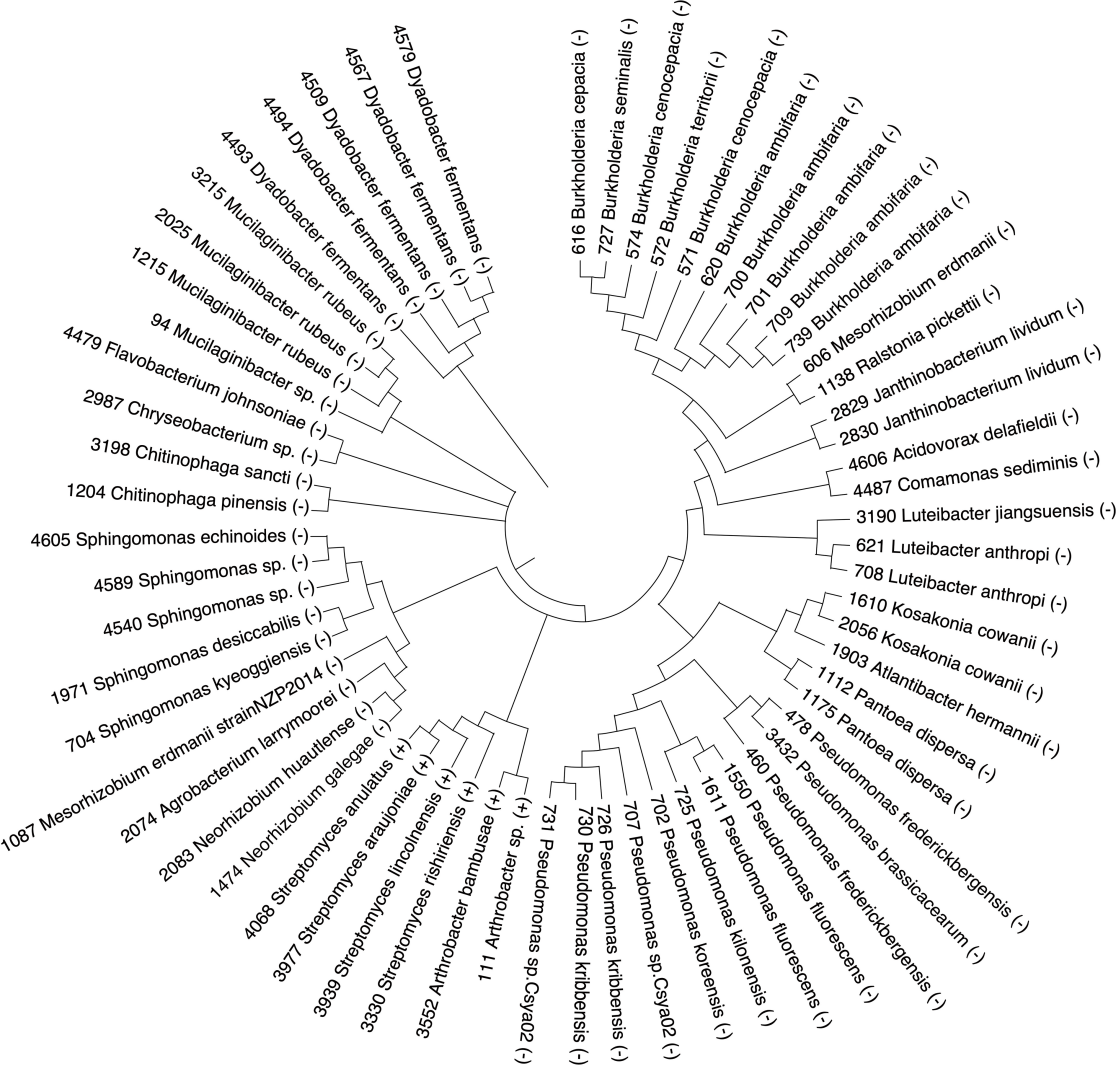
Phylogeny of the 63 bacterial taxa that were identified in a culture collection by searching with the ASVs identified in (Meier et al., 2022). Numbers that precede the genus on the phylogeny correspond to bacterial isolate numbers in our culture collection. Gram-negative bacterial isolates are indicated by the (-) at the end of the bacterial isolate name. Gram-positive bacterial isolates are indicated by the (+) at the end of the bacterial isolate name.

**Figure 1** was created by BioRender.com. **Figure 2** through 6 were prepared using the *R* packages “tiff” (Urbanek and Johnson, 2023), “gridExtra” (Auguie and Antonov, 2017), “readr” (Wickham et al., 2024), “ggplot2” (Wickham, 2016), and “magick” (Ooms, 2025).

## 3 Results

### 3.1 Identification of 63 potentially beneficial bacterial isolates

Previous work (Meier et al., 2022) identified amplicon sequence variants (ASVs) of soil microbes from the rhizosphere soil of 240 maize inbred lines that may contribute to increasing plant fitness under low N field conditions. These ASVs were associated with maize alleles that had undergone negative or positive selection. This suggested that these host alleles may have arisen in relationship to specific microbial groups by removing harmful alleles or increasing the frequency of desirable alleles. Therefore, we used an axenic growth system to test whether microbial taxa related to these ASVs, identified under low N conditions, impart a beneficial phenotype to maize. Initially the ASVs identified from field studies (Meier et al., 2022) were used to search 16S sequences from an in-house culture collection of over 2,000 isolates collected from soil, roots, and rhizospheres of multiple plant species collected in soils from across the state of Nebraska, USA. The outcome of that search using the 724 ASV sequences identified 63 bacterial isolates that had 95 – 100% similarity to the V4 region (**Supplementary Table 2**). The 63 bacterial isolates were tested to determine whether they imparted a growth advantage for maize under low N conditions. These isolates included many from the genus *Pseudomonas, Streptomyces, Burkholderia and Sphingomonas* (**Figure 2**). We also identified isolates from *Luteibacter, Dyadobacter, Chitinophaga, Mucilaginibacter, Pantoea, Janthinobacterium*, and *Kosakonia*. Most of these species are gram-negative except for 7 gram-positive taxa that included *Streptomyces* and two isolates of *Arthrobacter*.

### 3.2 Screening of the 63 potentially beneficial bacterial isolates

**Figure 3:**
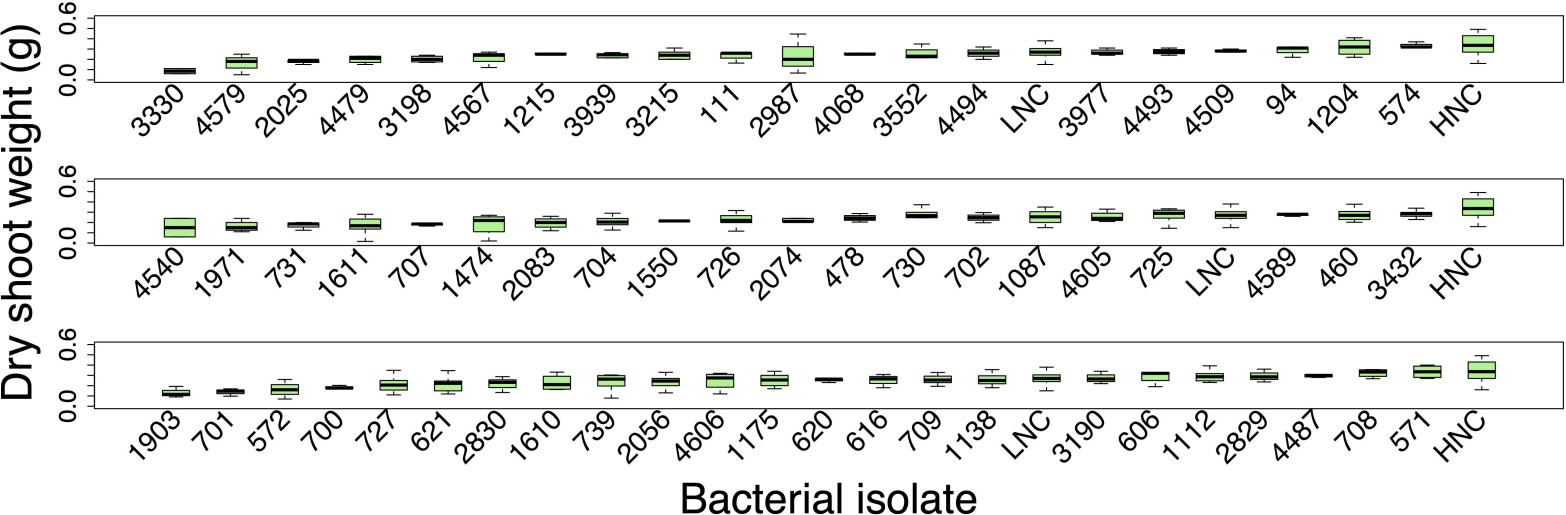
The dry shoot weight (g) results of the screening of the 63 potentially beneficial bacterial isolates. The plants were harvested at 14-days of growth when they were approximately at the V2 growth stage. Significant differences were determined using an ANOVA analysis followed by Tukey’s HSD correlation for multiple comparisons. HNC and LNC represent the high N control and low N control plants, respectively. Box plots display the median as the line within the box, as well as the lower quartile (bottom of the box) and the upper quartile (top of the box), with the whiskers showing the minimum and maximum data values). Each group is organized from lowest to highest median weight.

The screening (n = 3–8 plant per isolate) did not identify any of the bacterial isolates that significantly increased the fresh weight or dry shoot weight of the inoculated plants compared to the uninoculated low N control plants (**Figure 3**).

### 3.3 14-day validation experiment

Using the information from the screening, 15 bacterial isolates were selected based on increases in growth imparted to Mo17 under low N conditions (**Table 1**). These were then reinoculated to fresh Mo17 seeds and grown for 14 days under axenic conditions with more replicates (n = 7-8) to increase the statistical power compared to the screening. To select the 15 bacterial isolates for the 14-day experiment, the box plots of the screening results were analyzed visually to select bacterial isolates that appeared to increase the dry shoot weight.

**Table 1:** List of the 15 bacterial isolates selected from the screening for further testing in the 14-day validation experiment.

Bacterial isolate ID	Isolate identity	Similarity of ASV to sequence in culture collection (%)
111	*Arthrobacter* sp.	99.658
571	*Burkholderia cenocepacia*	99.315
574	*Burkholderia cenocepacia*	99.315
606	*Mesorhizobium erdmanii*	100
702	*Pseudomonas koreensis*	100
708	*Luteibacter anthropi*	99.315
726	*Pseudomonas kribbensis*	100
730	*Pseudomonas kribbensis*	100
1138	*Ralstonia picketti*	100
1204	*Chitinophaga pinensis*	96.575
2829	*Janthinobacterium lividium*	97.26
4487	*Comamonas sediminis*	97.603
4509	*Dyadobacter fermentans*	98.288
4589	*Sphingomonas* sp.	95.89
4606	*Acidovorax delafieldii*	98.63

**Figure 4:**
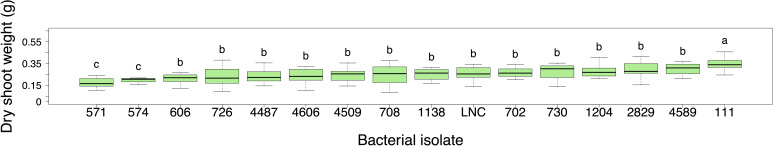
Dry shoot weight (g) of the Mo17 maize genotype inoculated with 15 selected bacterial isolates during the 14-day validation experiment. The plants were harvested at 14 days of growth, at approximately the V2 stage. A linear mixed model analysis was performed to assess the differences among treatments and the low N control. (Box plots display the median as the line within the box, as well as the lower quartile (bottom of the box) and the upper quartile (top of the box), with the whiskers showing the minimum and maximum data values. LNC represents the low N control plants. Letters above boxes represent statistically significant groupings (p < 0.05) different from the LNC). The isolates are arranged along the x-axis based on the lowest to highest median weight of the Mo17 plants. (n = 7-8, exception: n = 32 for LNC).

The plants were grown for 14 days and harvested at approximately the (V2) stage which was when the second leaf was fully expanded. In the 14-day validation experiment, bacterial isolate 111 (*Arthrobacter* sp.) significantly increased the dry shoot weight of Mo17 maize compared to the uninoculated low N control (p = 0.0013) (**Figure 4**). Two other bacterial strains improved maize seedling growth with relatively low variation including isolate 730 (*Pseudomonas kribbensis*), and 4589 (*Sphingomonas* sp.), but the increase was not statistically significant. The remainder of the bacterial isolates had no significant growth-promoting effect on the dry shoot weight compared to the uninoculated low N control.

### 3.4 Non-repeated inoculation experiment

From the results of the 14-day validation experiment, three bacterial isolates were selected for a 35-day experiment to assess the effects of these potentially beneficial bacterial isolates over a longer growth period when grown in pots in ambient air. These were isolate 111 (*Arthrobacter* sp.), 730 (*Pseudomonas kribbensis*), and 4589 (*Sphingomonas* sp.). Bacterial isolate 111 was selected due to its significant impact on the dry shoot weight of inoculated Mo17 maize compared to the uninoculated low N control (p-value = 0.0013) (**Figure 4**). Bacterial isolates 730 and 4589 were selected for their apparent ability to increase the Mo17 dry shoot weight under N-deficient conditions compared to uninoculated low-N control plants. In this experiment, we also tested these three isolates using three maize genotypes: Mo17, NSL 30867, and PI 606768 to determine whether there might be a maize genotype by bacterial isolate interaction. Thirty-five days after planting when the plants were approximately at the (V3 to V4) growth stage which was when the third or fourth leaf was fully expanded, the maize plants were harvested, and their dry shoot weights were measured.

In this experiment the bacterial isolates did not have a significant effect on the dry shoot weight (p = 0.7719) (**Table 2**), but the maize genotype did have a significant effect on the dry shoot weight (p < 0.0001) (**Table 2**) under N-deficient conditions. No significant interaction between bacterial isolate and maize genotype was observed (p = 0.3132) (**Table 2**).

**Table 2:** Non-repeated inoculation experiment – Linear mixed model analysis for the dry shoot weight (grams) response of each factor.

Effect	F-value	P-value
Bacterial Isolate	0.37	0.7719
Maize Genotype	39.35	< 0.0001
Bacterial Isolate x Maize Genotype Maize Genotype	1.2	0.3132

**Figure 5:**
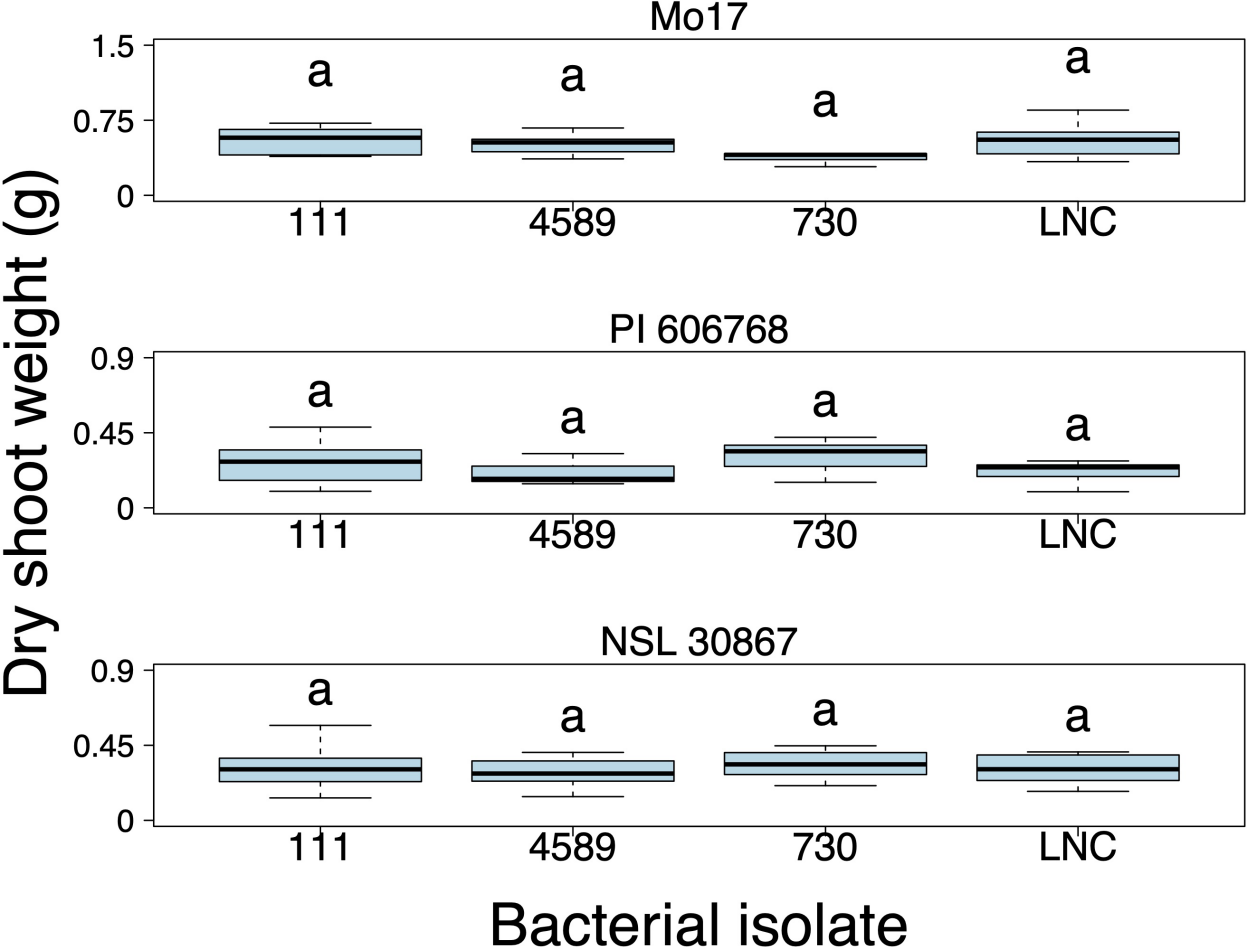
Dry shoot weight (grams) of the maize genotypes Mo17, PI 606768, and NSL 30867 inoculated with bacterial isolates during the non-repeated inoculation experiment. A linear mixed model analysis was performed to assess differences among treatments. Box plots display the median as the line within the box, as well as the lower quartile (bottom of the box) and the upper quartile (top of the box), with the whiskers showing the minimum and maximum data values. Letters above boxes represent statistically significant groupings (p < 0.05). LNC represents the low N control plants. (n = 9; Exceptions: n = 7 for LNC-PI 606768, n = 4 for 4589-PI 606768).

The impact of each bacterial isolate on the dry shoot weight of the inoculated plants at each specific maize genotype was further examined. However, we did not find that any of the three bacterial isolates significantly improved the growth of any of the maize genotypes grown under low N conditions (**Supplementary Table 3**) (**Figure 5**).

At the 14-, 21-, and 28 days after planting, images were collected of the plants to determine the aboveground biomass at different time points in the development of the plants. However, at none of the time points measured were there any significant differences in aboveground biomass between bacterial-inoculated plants and the uninoculated low N control plants (data not shown).

### 3.5 Repeated inoculation experiment

To determine whether a recurring soil drench of bacterial inoculant, as done in another study (Papin et al., 2025), may enhance the interaction between plant and bacterial isolate to reveal plant-growth-promoting abilities of the three selected bacterial isolates, a 28-day repeated inoculation experiment was carried out.

The dry shoot weight of the plants was measured 28 days after planting, when the plants were approximately at the V3-V4 stage. In this experiment the bacterial isolate alone did not have a significant effect on the dry shoot weight (p = 0.2047). (**Table 3**), but as in the previous experiment the maize genotype did have a significant effect on the dry shoot weight (p < 0.0001) (**Table 3**) under N-deficient conditions. Interestingly, there was a significant interaction effect between bacterial isolate and maize genotype (p = 0.0079) (**Table 3**), indicating that the dry shoot weight was dependent on the combined effect of the bacterial isolate and maize genotype. This result suggests that the bacterial isolate’s ability to act as a plant-growth-promoting species is dependent on the genetic makeup of the host plant.

**Table 3:** Repeated inoculation experiment – linear mixed model analysis for the dry shoot weight response of each factor.

Effect	F-value	P-value
Bacterial Isolate	1.57	0.2047
Maize Genotype	21.22	< 0.0001
Bacterial Isolate x Maize Genotype Maize Genotype	3.17	0.0079

**Figure 6:**
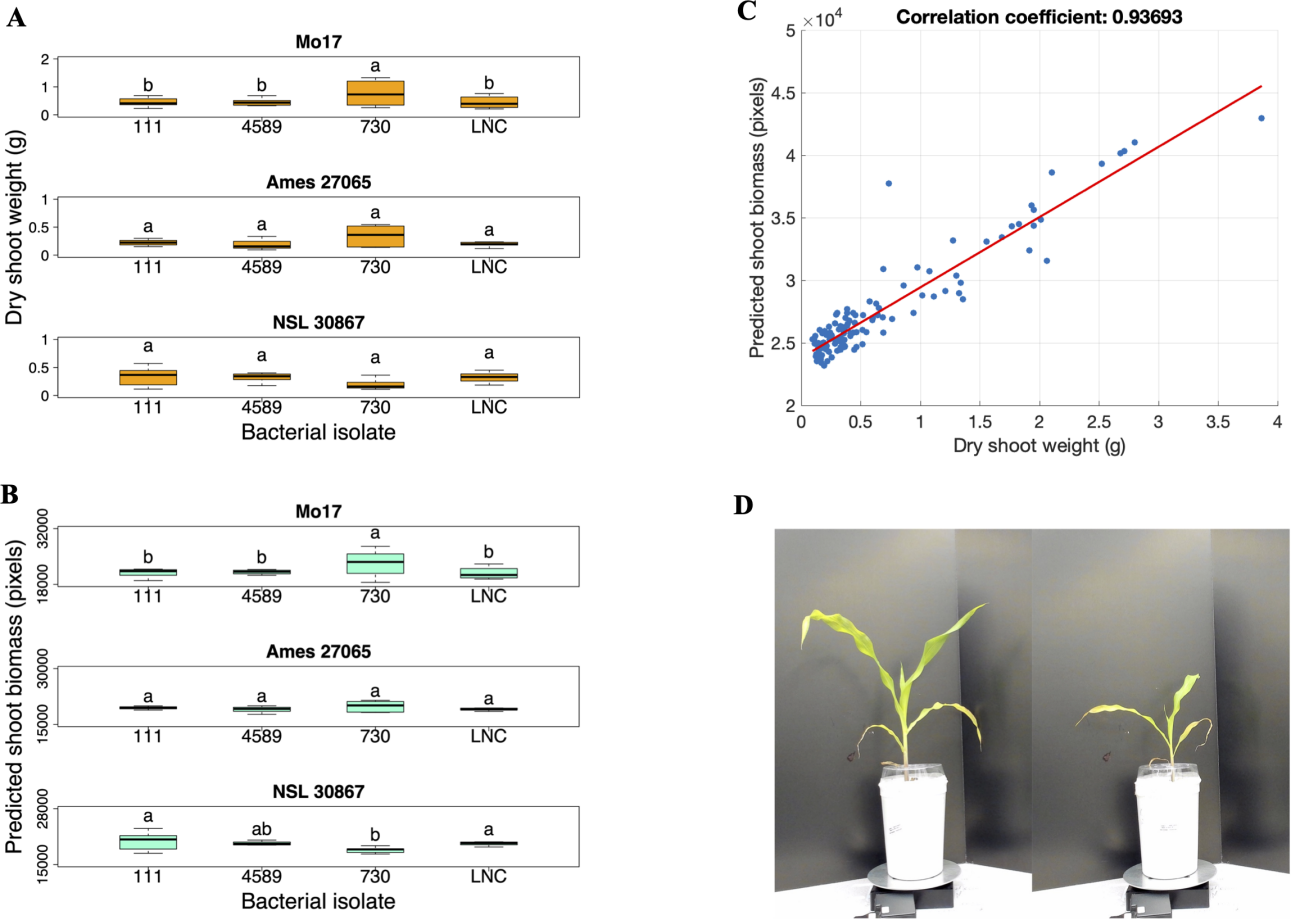
Repeated inoculation experiment. **(A)** Dry shoot weight (grams) and **(B)** Predicted biomass (pixels) of the Mo17, Ames 27065, and NSL 30867 maize genotypes inoculated with three bacterial isolates and the low N control (LNC). **(C)** Correlation analysis between the dry shoot weights of the 28-day old plants and the predicted shoot biomass based on image analysis at 27 days after planting (r = 0.937). **(D)** Two photos taken of 27-day-old, uninoculated Mo17 maize plants grown in high N (left) and low N (right) conditions in the repeated inoculation experiment. Different letters above the boxes indicate statistically significant differences at p < 0.05. LNC represents the low N control plants. Box plots display the median as the line within the box, as well as the lower quartile (bottom of the box) and the upper quartile (top of the box), with the whiskers showing the minimum and maximum data values. (n = 8; Exceptions: n = 9 for 730-NSL30867, n = 9 for LNC-NSL 30867, n = 7 for LNC-Ames 27065, and n = 6 for 730-Ames 27065.

Due to the significant interaction between maize genotype and bacterial isolate (**Table 3**), the effect of each bacterial isolate on the dry shoot weight was analyzed for each maize genotype (**Supplementary Table 4**). Bacterial isolate 730 had a significantly greater effect on dry shoot weight in the Mo17 maize genotype compared to the uninoculated low N control (p-value = 0.0008) (**Supplementary Table 4**; **Figure 6A**). In contrast neither isolates 111 or 4589 had a significant positive growth impact on the Mo17 (**Supplementary Table 4**; **Figure 6A**). Conversely, in the maize genotypes Ames 27065 and NSL 30867, none of the bacterial isolates had a significant growth effect on the dry shoot weight compared to the low N uninoculated control (**Supplementary Table 4**; **Figure 6A**). Inoculation with 730 (*Pseudomonas kribbensis*) resulted in a significant increase in dry shoot weight of Mo17 maize plants but not Ames 27065 or NSL 30867 which highlights the strong bacterial isolate host genotype interaction.

Images were captured at 14, 21, and 27 days after planting to determine whether growth increases could be measured at earlier time points than the final destructive harvest. The aboveground biomass derived from the imaging system (day 28) was correlated to the predicted biomass computed from images (day 27). The correlation coefficient was (r = 0.937) (**Figure 6B**), indicating good correspondence between measured and predicted biomass. At 14 days after planting, when the plants were approximately at the V2 stage, there was no significant increase in biomass of the bacterial-inoculated plants compared to the uninoculated low-N control plants. At 21 days after planting, when the plants were approximately at the V2-V3 stage, there was a significant interaction effect between the bacterial isolate and maize genotype (p = 0.0014) (**Table 4**), indicating that at 21 days after planting, specific bacterial isolates influenced the growth of certain maize genotypes, highlighting a specific host-microbe relationship. The effect of each bacterial isolate was determined for each maize genotype (**Supplementary Table 5**). We found there was a significant increase in growth of the N-deficient Mo17 inoculated with bacterial isolate 730 (*Pseudomonas kribbensis*) compared to the uninoculated N-deficient Mo17 maize (p-value = 0.0006) (**Figure 6C**; **Supplementary Table 5**) at day 21. Surprisingly, we also found that NSL30867 inoculated with 730 showed a significant decrease in growth at 21 days after planting, but not at 28 days when dry shoot biomass was measured. Representative images are shown of plants grown under nitrogen-deficient and nitrogen-sufficient conditions (**Figure 6D**).

**Table 4:** Repeated inoculation experiment (21-day image analysis) – linear mixed model analysis for the optically computed aboveground biomass response of each factor.

Effect	F-value	P-value
Bacterial Isolate	0.79	0.5058
Maize Genotype	23.49	< 0.0001
Bacterial Isolate x Maize Genotype Maize Genotype	4.05	0.0014

## 4 Discussion

### 4.1 Low success may be due to incorrect isolate identification

This study aimed to leverage plant genetic data and field-based 16S sequences from the maize rhizosphere to find isolates in a culture collection that enhance plant growth in low N settings. To accomplish this, we analyzed ASVs from the rhizosphere of maize cultivated in the field, which showed positive or negative links to alleles from a diverse group of maize lines (Flint-Garcia et al., 2005) in low N conditions (Meier et al., 2022). This was done to identify bacterial isolates from a laboratory culture collection containing more than 2,000 isolates. The ASVs were used to search the full or partial sequences of the 16S rRNA gene derived from each sequenced isolate in the collection. Others have identified growth-promoting bacterial species that improve maize and wheat growth, with some of these having N fixation properties (Shaharoona et al., 2008; Hungria et al., 2010; Alves et al., 2015), but there are few instances where plant genetic information has been used to identify bacterial taxa that enhance cereal plant growth. Our reasoning was that these ASVs, derived from culture-independent data associated with plant alleles that improve growth under low N conditions, would lead to the identification of unique isolates in a large culture collection that promote growth under low N conditions. From this search, we identified 63 isolates with a 95-100% match to the 16S ASV sequences from a culture-independent study. Upon further testing of the isolates on the maize genotype Mo17, only two isolates, 111 (*Arthrobacter* sp.) and 730 (*Pseudomonas kribbensis*), promoted the growth of Mo17 maize under low N conditions in a growth chamber. Bacterial isolate 730 belongs to the *Pseudomonas* genus and plant-growth-promoting properties of this genus have been documented, including phytohormone production, N fixation, siderophore production, and phosphate solubilization (Panpatte et al., 2016). In contrast the bacterial isolate 111 belongs to the *Arthrobacter* genus, and it has been reported that this genus possesses plant-growth-promoting properties, such as the ability to produce indole-3-acetic acid, solubilize phosphate, and fix N (Jiang et al., 2022). The specific plant-growth-promoting properties of 730 and 111, as well as the biological mechanisms enabling the maize genotype and bacterial inoculant interaction, remain to be further investigated. Our search revealed 63 isolates that matched the 16S ASV sequences from a culture-independent study with 95-100% accuracy. Further testing on the maize genotype Mo17 showed that only two isolates, 111 (*Arthrobacter* sp.) and 730 (*Pseudomonas kribbensis*), enhanced the growth of Mo17 maize in low N conditions within a growth chamber. The low success rate of this method in identifying growth-promoting isolates using field data may be attributed to a few factors. Initially, while the ASV sequences pinpointed culture collection isolates with up to 100% sequence identity, a key factor reducing the success rate is that numerous genes in bacterial genomes may show variation. A striking illustration of this comes from research on 19 *Pseudomonas fluorescens* strains collected from either the endosphere or rhizosphere of Eastern cottonwood (*Populus deltoides*). Despite these strains having 99% similarity in their 16S rRNA genes, notable differences were observed in the rest of the genome and in the phenotypes of the bacterial strains (Timm et al., 2015). The 16S rRNA gene is frequently utilized for phylogenetic analysis, but this approach often lacks taxonomic resolution below the genus level (Hartmann et al., 2019).

Other studies have used different approaches to identify growth-promoting bacteria. In one study, 474 bacterial isolates were obtained from the durum wheat rhizosphere and initially screened based on functional activities that may enhance plant acquisition of nutrients (Di Benedetto et al., 2019). In a first round of screening the pool of wheat rhizosphere bacteria was narrowed down to 333 and then in the second-round testing for mineralization of phosphate, production of IAA and nitrification, 16 isolates were identified as having potential plant growth promoting properties. Eventually three isolates with properties that warranted field testing were identified. Another study sampled 61 endophytic bacteria isolated from the leaves and stems of Pistachio (*Pistacia vera*). Through an initial screening, ten were identified as having unique functional properties and eight of the ten were identified as gram-negative. The next assessment was done based on the ability to promote root growth (Etminani and Harighi, 2018) and eight out of ten tested promoted root formation. Our study used field-derived ASVs that identified 63 isolates that were between 95 – 100% similarity to 16S sequences from a large collection of bacterial isolates. No other pre-screening tests were conducted to identify whether the bacterial isolates possess plant-growth-promoting capabilities, although testing for specific plant-growth-promoting factors may have been beneficial. The genetic approach used here was novel and identified at least two bacterial isolates that promote growth under different types of growth modalities. An improvement of this approach would have been to focus on culturing from the rhizosphere communities from which the original data was derived (Meier et al., 2022). The culture-independent microbiome-based data set with specific culturing of bacteria from the same samples would be the optimal approach that may improve the success rate in identifying growth-promoting bacteria.

### 4.2 Low success may be due to a lack of community

Another reason for the low success rate may be that each of the 63 bacterial isolates was inoculated individually and grown under axenic conditions without being given the opportunity to interact with other bacterial species. Cooperativity between members of a bacterial community may have been essential for observing growth-promoting effects. For example, some of the isolates can act as keystone species or assist in biofilm formation (Noirot-Gros et al., 2018), and others may recruit the beneficial bacteria to the rhizosphere (Liu et al., 2021). Within a rhizosphere community, bacterial species communicate with both the host plant (inter-kingdom communication) and with one another (intra-kingdom communication). Quorum sensing is one method of communication amongst rhizobacteria (Singh et al., 2022) that is important for biofilm formation (Hammer and Bassler, 2003; De Kievit, 2009). Auto-inducers are the signal molecules that bacterial species use in quorum sensing to communicate with other bacterial species (Schikora et al., 2016). Small, low-molecular-weight volatile organic compounds (VOCs) are also used to communicate within bacterial communities (Netzker et al., 2020) and these have been shown to promote the growth of other microbial species (Scott et al., 2019), and act directly as plant-growth promoting compounds (Park et al., 2015; Zhou et al., 2016).

Plants release exudates into the soil around their roots to influence the rhizospheric bacterial community members (Bertin et al., 2003). From there, recruited bacterial members interact with one another through cooperation or competition, influencing the plant’s overall health (Chepsergon and Moleleki, 2023). This may be important in the host-specific bacterial communities found within the rhizosphere and endosphere (Trivedi et al., 2020). Removing the dynamic relationships between bacterial species within a rhizosphere bacterial community in sterilized growth medium may also eliminate the beneficial interactions that promote the growth of the host plant. In various plant growth studies investigating the use of single bacterial isolates versus a consortium isolates, using a multi-isolate inoculum enhanced plant growth more than a single-isolate inoculum. For example, when inoculated together, *Bacillus velezensis* and *Pseudomonas stutzeri* had a beneficial growth effect on plant traits compared to plants inoculated with only one species (Sun et al., 2022). This was thought to be because the *B. velezensis* stimulated *P. stutzeri*, forming biofilms on the plant root surface, indicating cooperation between the two inoculated species. Similarly, PGPRs combined and inoculated on maize performed better than each PGPR inoculated individually (Molina-Romero et al., 2017). Moving forward, it will be necessary to simultaneously test the inoculation of multiple members of the 63 isolates or use non-sterile soils that contain native communities and would be more relevant to agroecosystems.

### 4.3 Repeated inoculations or very high propagule numbers are essential for growth promotion

In this study, we found that repeated inoculation of a bacterial isolate was more beneficial for enhancing plant growth with certain isolates than one or two introductions of a bacterial strain. When a novel organism is introduced to an environment, propagule pressure is a key factor in colonization success that must be considered. Propagule pressure is a concept that accounts for the frequency of introduction events as well as the number of individuals per event that are introduced (Koontz et al., 2018). This concept is often discussed in invasion biology (Stringham and Lockwood, 2021); however, propagule pressure may also be applied to help formulate the most effective bacterial inoculation strategies to maximize the plant growth-promoting effects of the bacterial species. In this study, we found that bacterial isolate 730 (*Pseudomonas kribbensis*) significantly increased the dry shoot weight of maize grown under N-deficient conditions under a repeated inoculation protocol. We did not find the same result in our non-repeated inoculation experiment. In the repeated inoculation experiment, six repeated inoculations were carried out, compared to the two inoculations done in the non-repeated inoculation experiment. Similar to our findings, others have shown that repeated inoculations promote plant growth in maize (Papin et al., 2025) and wheat (Chen et al., 2024), whereas one to two inoculations do not. The timing of inoculations may also be important. In one study, repeated inoculations in soil *prior* to maize sowing promoted the growth of aboveground biomass, while repeated inoculations carried out *after* maize sowing impacted the resident bacterial community but did not impact maize growth (Papin et al., 2025). In contrast, an increase in wheat growth as well as an increase in both soil bacterial diversity and functional genes related to the N cycle was reported (Chen et al., 2024) following repeated bacterial inoculations performed after sowing.

In our study, it was also noteworthy that the beneficial effects of bacterial isolate 730 (*Pseudomonas kribbensis*) were significant at the 21-day timepoint in the repeated inoculation experiment, but not at the 14-day timepoint. Time was not included as a variable in our statistical analysis, but it is clearly important in experimental design, as short-term experiments may be less useful in determining the growth-promoting effects of bacterial isolates. The plant growth-promoting effects of various bacteria, like *Bacillus*, have been reported as significant at the V6 growth stage of maize, but not at the V4 growth stage (Lin et al., 2019). In contrast to the results of the repeated inoculation experiment, bacterial isolate 111 (*Arthrobacter* sp.) significantly promoted the growth of Mo17 maize after just 14 days, but the growth promotion was not observed after that time point. Similarly, an *Arthrobacter nicotinovorans* strain isolated from the rhizosphere of *Panax ginseng* was found to significantly increase the shoot weight of inoculated ginseng plants after 15 days of growth (Jiang et al., 2022). Therefore, the experimental timing, multiple inoculations and the application of nondestructive phenotyping methods utilized here are important considerations when studying plant growth promotion by bacteria.

### 4.4 Plant genotype is essential for action of growth promotion

Plant genotype is known to influence the composition of the bacterial communities in roots, rhizosphere, and the phyllosphere (Aira et al., 2010; Bodenhausen et al., 2014; Marques et al., 2014). Two of the well-known mechanisms that shape plant bacterial interactions include the immune system (Tzipilevich et al., 2021) and root exudates (Sasse et al., 2018). It has also been shown that maize genotypes (Lopes et al., 2022; Wang et al., 2022; Lopes et al., 2023a) influence the composition of microbial communities through the release of different amounts of root exudates composed of sugars, amino acids, organic acids, and phenolic compounds (Coskun et al., 2017). Host genetics has also been demonstrated to have a strong influence on the diversity and composition of microbiomes, but less is known about the mechanisms behind the association of plant genes with microbial community structure (Peiffer et al., 2013; Wagner et al., 2016; Gehring et al., 2017). For example, when investigating the genetic variation among 302 natural accessions of *Arabidopsis thaliana* plants, researchers reported there were certain candidate genes, involved in important plant growth-related processes, that were also highly associated with how well the accession responded to the PGPR effects of *Pseudomonas simiae* WCS417r (Wintermans et al., 2016). Likewise, in another study, it was reported that the interaction between PGPB and the maize genotypes in 360 lines of tropical maize germplasm was controlled by multiple genes within the maize genotypes (Yassue et al., 2021).

Although there are interactions between members of bacterial communities and plant genotypes, there are few clear examples of this in the literature (Vargas et al., 2012; Do Amaral et al., 2016). Genotype by bacterial isolate interactions may be a critical factor limiting the widespread successful application of bacterial growth-promoting inoculants in agriculture. In a previous study, we showed genotypic differences in sorghum growth responses to a cocktail of 10 bacterial isolates (Chai et al., 2021) under low N conditions. In this current study, we found that one bacterial isolate from the genus *Pseudomonas* (730) only enhanced the growth of one (Mo17) and not two other maize genotypes. The other two genotypes were chosen based on total sugars and jasmonic acid in their exudates (Lopes et al., 2022). We hypothesized that because the concentrations of total sugars and jasmonic acid in the exudates impacted the rhizospheric bacterial communities in the maize plants (Lopes et al., 2022), perhaps the plant-growth-promoting effects of the bacterial isolates 111, 730, and 4589 would also be influenced by the exudate concentrations. In the repeated inoculation experiment of our study, there was a significant interaction (p = 0.0079) between the maize genotype and the bacterial isolate, indicating that maize genotypes respond differently to the plant-growth-promoting bacterial isolates tested in this study.

## 5 Conclusion

Our research emphasizes key factors to consider when screening soil bacteria that promote plant growth. We demonstrated that both the number and potentially the timing of inoculation significantly influence a bacterial isolate’s ability to enhance plant growth. We also confirmed the hypothesis that plant host genotype is critical to the development of positive bacterial interactions and suggest that other microbial community members are essential elements when testing inoculants under controlled conditions. Although our method of using genetically identified ASVs to select culture collection isolates did not yield a significant number of growth-promoting isolates, it is possible that culturing directly from the field-derived rhizosphere samples could have improved the success rate. In future, considering these multiple factors may significantly enhance the success rate of identifying growth-promoting bacterial isolates and may also lead to increased reliability and repeatability of biostimulants across a broader range of agroecosystems.

## Data Availability

The datasets presented in this study can be found in online repositories. The names of the repository/repositories and accession number(s) can be found in the article/**Supplementary Material**.
